# Heme Oxygenase-1 as Therapeutic Target for Diabetic Foot Ulcers

**DOI:** 10.3390/ijms231912043

**Published:** 2022-10-10

**Authors:** Ermelindo Carreira Leal, Eugenia Carvalho

**Affiliations:** 1 Center for Neuroscience and Cell Biology, University of Coimbra, 3004-504 Coimbra, Portugal; 2 Institute of Interdisciplinary Research, University of Coimbra, 3004-504 Coimbra, Portugal

**Keywords:** heme oxygenase-1, diabetes, diabetic foot ulcers, wound healing, skin, oxidative stress, inflammation

## Abstract

A diabetic foot ulcer (DFU) is one of the major complications of diabetes. Wound healing under diabetic conditions is often impaired. This is in part due to the excessive oxidative stress, prolonged inflammation, immune cell dysfunction, delayed re-epithelialization, and decreased angiogenesis present at the wound site. As a result of these multifactorial impaired healing pathways, it has been difficult to develop effective therapeutic strategies for DFU. Heme oxygenase-1 (HO-1) is the rate-limiting enzyme in heme degradation generating carbon monoxide (CO), biliverdin (BV) which is converted into bilirubin (BR), and iron. HO-1 is a potent antioxidant. It can act as an anti-inflammatory, proliferative, angiogenic and cytoprotective enzyme. Due to its biological functions, HO-1 plays a very important role in wound healing, in part mediated through the biologically active end products generated by its enzymatic activity, particularly CO, BV, and BR. Therapeutic strategies involving the activation of HO-1, or the topical application of its biologically active end products are important in diabetic wound healing. Therefore, HO-1 is an attractive therapeutic target for DFU treatment. This review will provide an overview and discussion of the importance of HO-1 as a therapeutic target for diabetic wound healing.

## 1. Introduction

The development of chronic non-healing diabetic foot ulcers (DFU) is one of the most serious and costly complications of diabetes, with high recurrence rates, causing a significant burden to patients [1,2]. The DFU physiopathology is complex since diabetes-induced low-grade inflammation, peripheral neuropathy, peripheral vascular disease, and infection foster tissue damage and often can lead to amputations [3,4]. It has been estimated that the risk of developing a DFU in a patient’s lifetime is as high as 25%, with a high percentage of these leading to amputation [1,5,6,7]. The pathogenesis of chronic non-healing DFUs is not fully understood; therefore, the development of effective treatment strategies is very challenging.

The biological processes involved in wound healing are divided into four overlapping phases: hemostasis, inflammation, proliferation, and remodeling [4]. However, under diabetic conditions, these healing phases become stalled, particularly in the early inflammatory phase, predisposing patients to chronic diabetic foot ulcers [8]. In contrast to normal wounds, diabetic wounds are characterized by persistent oxidative stress and inflammation [9,10], with increased expression of pro-inflammatory cytokines and infiltration of inflammatory cells [10,11]. The increase in inflammatory cells at the wound site under diabetic conditions is associated with the overproduction of reactive oxygen species (ROS), causing significant tissue damage [12]. In addition, the excessive ROS impair angiogenesis, cell migration and proliferation, as well as the degradation of the extracellular matrix (ECM) [10,13,14,15]. Thus, it is evident that targeting inflammation and oxidative stress could be an important strategy in improving impaired healing in diabetic subjects. Heme oxygenase-1 (HO-1) is an important defense mechanism against cellular stress, promoting antioxidant defense and enhancing cell survival [16,17]. HO-1 is an inducible enzyme that degrades heme into carbon monoxide (CO), biliverdin (BV), which is consequently reduced to bilirubin (BR) by biliverdin reductase, as well as iron [18]. HO-1 is induced by natural phytochemicals, statins, metals [19,20,21], and also by several other stimuli that include oxidative stress, inflammation, hyperoxia, hypoxia, and wounded tissues [22,23]. The products of HO-1 activity, particularly CO, BV, and BR, are not only antioxidant and antiapoptotic molecules but they also promote other functions, such as anti-inflammation and angiogenesis, which are important for wound healing [24,25,26,27,28,29]. Additionally, iron derived from HO-1 activity induces the synthesis of ferritin, which sequesters the released iron and limits the potential of iron to catalyze the formation of cytotoxic products [30]. Since several studies have shown that HO-1 holds antioxidant, anti-inflammatory, cytoprotective, proliferative, and angiogenic properties [31,32,33], it is clear how HO-1 plays a key role in the wound healing process [26,34].

Therefore, the present review will further examine the important role of HO-1 in diabetic wound healing and discuss the therapeutic potential of HO-1 induction for the treatment of chronic non-healing DFU. 

## 2. Normal Wound Healing vs. Chronic Non-Healing Diabetic Wounds

The wound healing process is composed of four very well synchronized and overlapping phases: hemostasis, inflammation, proliferation, and remodeling [4]. Immediately after skin injury, in the hemostasis phase, the blood vessels constrict to restrict blood flow, and the platelets are activated to stop the loss of blood by promoting the formation of a clot at the site of vessel rupture. Platelets initiate the inflammatory phase by releasing factors that attract immune cells from the circulation into the local wound site. Neutrophils are attracted to the wound site, followed by monocytes that in turn differentiate into macrophages in the local tissue environment [35]. Neutrophils at the wound site produce high levels of ROS, proteases, and pro-inflammatory cytokines that help prevent infections. Then, neutrophils apoptosis occurs and macrophages phagocyte the death cells, cleaning up the local wound site [36]. Macrophages are key regulators of the wound healing process. These cells change their phenotype along the wound progression [10,37,38]. Early in the inflammatory phase, pro-inflammatory macrophages, usually referred to as M1 macrophages, infiltrate to clean the wounded tissue of dead cells, debris, and bacteria. At the beginning of the proliferative phase, in normal wounds, most of the macrophage population adopt an anti-inflammatory and regenerative phenotype, usually referred to as M2 macrophages. The M2 macrophages promote the migration and proliferation of fibroblasts, keratinocytes, and endothelial cells. In the proliferative phase, endothelial cells, fibroblasts, and keratinocytes proliferate and migrate to the wound site to regenerate the injured tissue. This phase is characterized for the formation of the granulation tissue, a loose vascularized tissue, with ECM produced by fibroblasts that provide oxygen and nutrients to promote the regeneration of the damaged tissue [39]. In the remodeling phase, macrophages release matrix metalloproteinases (MMPs) to breakdown the temporary ECM. The ECM then matures, reorganizing the fibers and increasing in mechanical strength [4]. Wound healing is complete following apoptosis of the extra proliferative cells, macrophages, myofibroblasts, and vascular cells, so the skin matures to the non-wounded state, with a collagen-rich scar remaining [10,15,36,39,40].

Oxidative stress plays a critical role in diabetic complications. In diabetes, ROS is highly produced by mitochondria due to the high levels of glucose oxidation [41]. ROS inhibits glyceraldehyde-3-phosphate dehydrogenase (GAPDH), leading to the accumulation of glyceraldehyde-3-phosphate, which is involved in the activation and formation of advanced glycation end products (AGEs) and in the formation of diacylglycerol production which, in turn, activates the PKC pathway [42,43]. Both pathways, AGEs and PKC, are activated and involved in the increased production of ROS [43]. Furthermore, in diabetic conditions, the accumulation of fructose-6-phosphate levels leads to an enhanced flux along the hexosamine pathway, increasing ROS levels [44]. Moreover, high glucose activates the polyol pathway, which decreases NADPH levels affecting the antioxidant system by depleting the levels of antioxidant glutathione (GSH). Oxidative stress due to high ROS levels can be aggravated with dysregulation of the antioxidant defense system in diabetes [45].

In chronic non-healing diabetic ulcers, hyperglycemia induces an increase in oxidative stress, as referred to previously, and dysregulates the function of neutrophils and macrophages, resulting in a persistent inflammatory state [9,10]. In addition, the increased number of neutrophils further increases the production of ROS during the inflammatory stage, damaging the cells at the wound site [10,11]. Hyperglycemic conditions drive macrophage polarization towards the pro-inflammatory M1 phenotype, which causes excessive and prolonged inflammation in diabetic wounds [9,10]. Therefore, wounds remain in the inflammatory phase, damaging the wounded tissue and inhibiting tissue regeneration [36]. In the proliferation phase, oxidative stress impairs endothelial cells, keratinocyte, and fibroblast, decreasing their proliferation, migration, and differentiation, as well as increasing the levels of secreted inflammatory factors, such as interleukin (IL)-8, produced by keratinocytes [46,47]. In the last phase, both collagen deposition and ECM remodeling are impaired due to the excessive oxidative stress levels and the persistent inflammatory environment [10,13,14,15]. Furthermore, chronic non-healing diabetic wounds are characterized by the production of high levels of matrix metalloproteinase (MMPs) and a significant decrease in growth factors, such as transforming growth factor 1 (TGF-1), which are important in tissue regeneration and angiogenesis [48,49]. Collagen production is also significantly decreased in diabetic wounds [15]. Moreover, diabetic wounds have high MMP activity, which inhibits the remodeling process and delays wound healing [50].

Taken together, the oxidative stress and inflammation induced by hyperglycemia impact all wound healing phases, including most of the cells present at the wound site as well as the mechanisms involved in wound healing. Targeting the molecular causes of sustained oxidative stress and inflammation in non-healing chronic wounds will be a powerful and effective strategy to promote diabetic wound healing.

## 3. Heme Oxygenase-1

**Figure 1:**
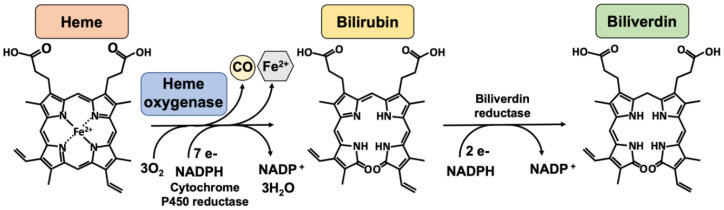
The heme oxygenase enzymatic reaction. Heme oxygenases degrade heme to sequentially generate carbon monoxide (CO), ferrous iron (Fe^2+^), and biliverdin. The reaction requires 3 mol of molecular oxygen and 7 electrons from NADPH-cytochrome P450 reductase. Bilirubin is subsequently reduced to bilirubin by an NADPH-dependent biliverdin reductase. Legend: NADPH, nicotinamide adenine dinucleotide phosphate.

Heme oxygenases consist of a family of enzymes responsible for heme (a pro-oxidant agent) catabolism, in a reaction that produces carbon monoxide, biliverdin, which is consequently reduced to bilirubin by biliverdin reductase, and iron (Figure 1) [18]. Two HO isoforms are responsible for this catalytic activity, an inducible enzyme, HO-1, and a constitutively active enzyme, HO-2. HO-3 is not well known and does not have enzymatic activity [51]. 

HO-1 is an ubiquitous stress protein, with a 32-kDa molecular weight, expressed in low quantities under normal conditions except in tissues that involve the degradation of senescent red blood cells, such as the spleen, liver, and bone marrow [51]. The expression of HO-1 can be induced in response to either exogenous or endogenous stimuli, including ultraviolet (UV) irradiation, natural phytochemicals, statins, heavy metals, heat shock, inflammatory stimuli, heme, oxidative stress, cobalt protoporphyrin-IX (CoPP), iron starvation, hyperoxia, and hypoxia [19,20,21,52]. Oxidative stress can activate signaling pathways such as the mitogen-activated protein kinases (MAPK), protein kinase C (PKC), 5′-AMP-activated protein kinase (AMPK), and phosphoinositide 3-kinase/protein kinase B (PI3K/Akt), which induce HO-1 expression through the transcription factor nuclear factor erythroid 2–related factor 2 (Nrf2) [53,54]. Nrf2 disassociates from its inhibitor, kelch-like erythroid cell–derived protein 1 (Keap1), translocating to the nucleus where it induces the expression of several key antioxidant genes including HO-1 [55]. Other transcription factors are known to induce HO-1 expression, including activator protein-1 (AP-1), signal transducer and activator of transcription 3 (STAT-3), Yin Yang 1 (YY1), and hypoxia inducible factor (HIF)-1 alpha, through stimuli such as oxidative stress, hypoxia, heme, and IL-6 [53,54]. On the other hand, BTB and CNC homology 1 (Bach)1 is a transcription factor which inhibits HO-1 expression [24]. Moreover, HO-1 is inhibited by metalloporphyrins, including tin protoporphyrin-IX (SnPPIX) and zinc protoporphyrin-IX (ZnPPIX), which compete with heme for the HO-1 binding site [56,57]. The metalloporphyrins, based in the porphyrin structure, have been tested for their ability to competitively inhibit the degradation of the heme group. Both SnPPIX and ZnPPIX have been shown to strongly inhibit heme degradation. However, SnPPIX was found to be the most potent inhibitor of HO activity [58]. A list of modulators of HO-1 biosynthesis and inhibitors of the enzymatic activity of HO-1 are represented in Table 1.

HO-1 is known to be localized in the endoplasmic reticulum, anchored via the COOH terminus. In the endoplasmic reticulum, HO-1 is in the proximity of cytochrome P450 reductase, which is required for its high enzymatic activity [59,60]. Furthermore, HO-1 has been observed in other cellular localizations such as in caveolae, mitochondria, and in nucleus, which has been associated with the truncation of the COOH terminus and loss of enzymatic activity, suggesting that HO-1 may have other bioactive actions other than catalyzing heme degradation [61,62].

The effects of HO-1 in tissue protection are evident in patients who have HO-1 genetic deficiencies, with accumulation of heme and iron, leading to increasing inflammation and ROS. This in turn results in damage of the liver, kidney, and vasculature [63]. Moreover, mice with HO-1 deficiency present high levels of heme in circulation and concomitant increases in inflammation [64]. In addition, in several animal models of disease, overexpression of HO-1 levels has been shown to confer protection to the heart, lung, and vasculature, as well as against skin injury [34,65,66].

**Table 1:** Modulators of HO-1 biosynthesis and inhibitors of HO-1′s enzymatic activity.

	HO-1 Modulator	Model	Refs.
**HO-1 inducers**			
Oxidants	UV irradiation	Mouse	[67]
	H_2_O_2_	HaCat cells, Human primary melanocytes	[68,69]
	Menadione	Rat primary hepatocytes	[70]
	(superoxide donor)		
Inflammatory stimuli			
	LPS	Mouse LPS-induced septic shock	[71]
		RAW264.7 cells	[72,73]
	IL-6	HEPG2 cell	[74]
Metalloporphyrins			
	Heme	K562 cells	[75]
	Hemin	Diabetic rat wound healing	[26,76]
	CoPP	Aorta diabetic rats	[18]
Pharmacological agent			
	Statins	HT-29 cells	[20]
	Aspirin	Human primary melanocytes	[69]
Natural Phytochemical			
	Resveratrol	HaCat cells	[77]
	Curcumin	Human primary skin fibroblasts	[78]
	Quercetin	HDF cells, HEKC cells	[79]
	EGCG	BV2 microglia cells	[80]
Chemicals			
	CoCl2	HK-2 cells	[80]
	CdCl2	HK-2 cells	[81]
Physical stress			
	Heat stress	Heat stress-stimulated rat liver	[82]
Oxygen levels			
	Hypoxia	Mice overexpressing HO-1 in lungs	[66]
	Hyperoxia	Rats	[83]
Transcription factors			
	Nrf2	Diabetic rat wound model	[84]
	AP-1	Mouse model of sepsis	[73]
	STAT-3	HEPG2 cell	[74]
	YY1	Rat aortic smooth muscle cells	[85]
	HIF-1 alpha	UV irradiation in mice	[67]
Gene therapy			
	Adenovirus mediated HO-1 transduction	Mouse, systemic administration	[86]
		Rat primary cardiomyocytes	[87]
	HO-1 gene transfection	HEPG2 cell	[74]
		RBL2H3 cells	[88]
		Mouse primary keratinocytes	[34]
	HO-1 gene	Transgenic mice overexpressing HO-1 in keratinocytes	[34]
**HO-1 enzymatic inhibitors**			
Metalloporphyrins	SnPPIX	Diabetic rat wound healing	[26,33,76]
		Rat VSMC, RAW264.7 cells	
	ZnPPIX	Rat VSMC, RAW264.7 cells	[33]
**HO-1 repressor**			
Transcription factor	Bach-1	NIH/3T3 cells, murine embryonic fibroblasts and murine erythroleukemia cells	[89]

Abbreviations: activator protein-1 (AP-1), BTB and CNC homology 1 (Bach-1), cobalt protoporphyrin-IX (CoPPIX), epigallocatechin gallate (EGCG), lipopolysaccharide (LPS), human hepatoma cells (HEPG2 cell), human dermal fibroblasts cell line (HDF), human epidermal keratinocyte cell line (HEKC), human keratinocyte cell line (HaCat), human pro-erythroid cells (K562), human renal proximal tubular epithelial cell line (HK-2), hypoxia inducible factor 1 alpha (HIF-1 alpha), mouse embryo fibroblast cell line (NIH/3T3 cells), mouse macrophage cell line (RAW264.7), nuclear factor erythroid 2–related factor 2 (Nrf2), rat mastocytoma cell line (RBL2H3), human renal tubular epithelial cells (HK-2), signal transducer activator of transcription 3 (STAT-3), tin protoporphyrin-IX (SnPPIX), Yin Yang 1 (YY1), vascular smooth muscle cells (VSMC), zinc protoporphyrin-IX (ZnPPIX).

## 4. HO-1 in Wound Healing

Immediately after injury, HO-1 is induced in the wounded tissues [22,23]. During hemolysis, the pro-oxidant heme is released, triggering inflammation and oxidative stress [90]. Heme is a strong inducer of HO-1, which degrades heme and has an important protective role against the oxidative and inflammatory results in the local wounded tissue [22,90]. It is known that the effect of preventing heme toxicity by HO-1 is mediated by the activation of Nrf2 [75]. 

The importance of HO-1 in wound healing has been confirmed not only by using HO-1 deficient mice but also by pharmacological inhibition of HO-1, which results in a significantly delayed wound closure [34,90,91]. In both models, the wounds showed an increase in oxidative stress and inflammation, as well as impaired wound re-epithelialization and angiogenesis [34,90,91]. In addition, hemin, an inducer of HO-1, was able to significantly improve wound healing, acting as an anti-inflammatory agent [92]. 

HO-1 is also a key player in the regulation of diabetic wound healing, but the effect is limited due to the high basal levels of oxidative stress in diabetes that are responsible for the damage of proteins, lipids, and DNA, which can lead to tissue impairment. Under this condition, the increased oxidative stress and inflammation that further occurs after wounding can result in impaired wound healing. Diabetic skin tissue collected from peri-wound regions showed higher levels of oxidative stress markers than peri-wound skin regions from non-diabetic patients. Similarly, it has been shown that HO-1 levels are increased in rodent diabetic wounded tissues via Nrf2 activation. This may suggest a compensatory mechanism for the increase in oxidative stress, demonstrating the important contribution of HO-1 in diabetic wound healing [93]. 

Animal models of diabetes have also been used to better understand the role of HO-1 in diabetic wound healing. HO-1 levels are increased after wounding in non-diabetic and db/db mice, a type 2 diabetic animal model, particularly in the initial wounding phase [34]. However, the increase in HO-1 levels in diabetic wounds appears to be delayed, which may trigger the impaired wound process overserved under these conditions. Similarly, the induction of HO-1, using an intradermal injection of hemin, was impaired in diabetic mice, showing a decrease in HO-1 levels [34]. On the other hand, the local delivery of HO-1 transgene by adenoviral vectors improved wound healing in diabetic db/db mice by increasing neovascularization [34]. Furthermore, the HO-1 inducer hemin has been shown to promote wound healing in diabetic rats by reducing inflammatory cytokines, increasing antioxidant defenses, and promoting angiogenesis [26,76]. Hemin has been shown to decrease oxidative stress by an increase in the antioxidant defenses in several experimental models [94,95], and to decrease inflammatory cytokines in animal models of disease [94,96]. However, since the use of the HO-1 inhibitor SnPPIX, in the presence of hemin, has shown the opposite effect of hemin, delaying wound healing in diabetic rats, this suggests that the effects of hemin may be through the induction of HO-1 [26,76].

Based on these evidence, HO-1 has a significant impact on wound healing and may be beneficial in the treatment of chronic wounds. Studies showing the importance of HO-1 in wound healing in different experimental models are presented in Table 2.

## 5. The Biological Properties of HO-1 Promoting Wound Healing

**Figure 2:**
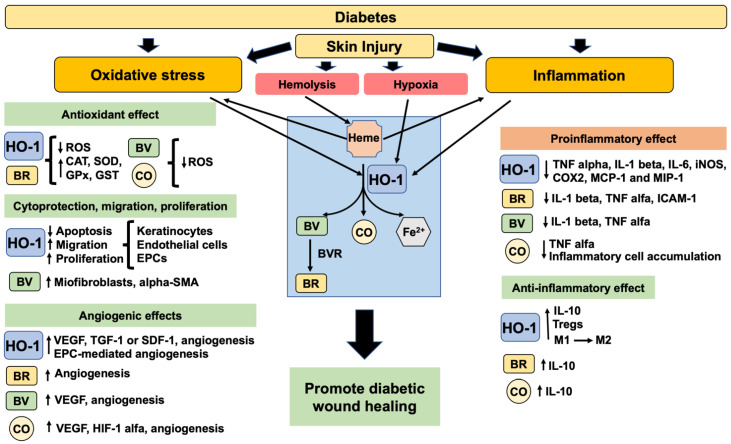
The beneficial role of HO-1 and its enzymatic products in promoting diabetic wound healing. Diabetes has an excessive oxidative stress and inflammation impairing the response to skin injury. After injury, the hemolysis will release heme groups (pro-oxidants) that will promote HO-1 induction. Also, the expression of HO-1 is induced by oxidative stress, inflammation, and hypoxia. HO-1 and the products of HO-1 enzymatic activity will decrease oxidative stress and proinflammatory factors. Moreover, they will promote the increase in cell viability, anti-inflammatory factors, migration, proliferation, angiogenesis and consequently improve diabetic wound healing. Legend: alpha-smooth muscle actin (alpha-SMA), anti-inflammatory macrophage M2 (M2), bilirubin (BR), biliverdin (BV), biliverdin reductase (BVR), carbon monoxide (CO), catalase (CAT), cyclooxygenase 2 (COX2), endothelial progenitor cells (EPCs), glutathione peroxidase (GPx), glutathione S-transferase (GST), heme oxygenase 1 (HO-1), hypoxia inducing factor 1 alpha (HIF-1 alpha), inducible nitric oxide synthase (iNOS), interleukin 1 beta (IL-1 beta), intercellular adhesion molecule-1 (ICAM-1), interleukin 6 (IL-6), interleukin 10 (IL-10), macrophage inflammatory protein-1 (MIP-1), monocyte chemoattractant protein 1 (MCP-1), proinflammatory macrophage M1 (M1), reactive oxygen species (ROS), stromal derived factor 1 (SDF-1), superoxide dismutase (SOD), transforming growth factor 1 (TGF-1), tumor necrosis factor alpha (TNF alpha), vascular endothelial growth factor (VEGF).

HO-1 is a cytoprotective enzyme that plays a role in wound healing through its antioxidant and inflammatory properties, as well as its influence on cell proliferation, migration, and angiogenesis. Here, we will discuss several studies showing that HO-1 properties are important to wound progression and the mechanisms of wound healing through HO-1 induction or inhibition. Moreover, we will discuss the effect of the products generated by the enzymatic activity of HO-1, known to have a beneficial role in wound healing, which are CO, BV, and BR, a precursor of BV. The beneficial role of HO-1 and its enzymatic products in promoting diabetic wound healing is represented in Figure 2.

### 5.1. Antioxidant Properties

Oxidative stress, which results from excessive production of ROS or a reduced antioxidant defense, may change the structure and chemistry of proteins, lipids, and nucleic acids. ROS enhances cellular damage, and it plays a role in the development of diabetes and its complications [98]. Persistent hyperglycemia contributes to the increase of ROS production, which aggravates oxidative stress. 

In wound healing, ROS levels are increased, playing a critical role in the activation of survival pathways and providing defense against invading microorganisms [99]. However, in diabetes, one of the main causes of non-healing chronic wounds is the excessive production of ROS and the decrease in antioxidant defenses [100]. HO-1 induction is crucial as a defense mechanism against cellular stress by its antioxidant properties [101], particularly in diabetic wounds, as the expression of other antioxidant enzymes is decreased, such as superoxide dismutase (SOD), glutathione peroxidase (GPx), glutathione-S-transferase (GST), or catalase [102]. In addition, in diabetic wounds, ascorbic acid, vitamin D, and glutathione (GSH), which act as antioxidant defenses, are significantly decreased [102]. 

Several studies have shown the effect of HO-1 regulation in diabetic-induced oxidative stress in wound healing. The use of hemin, a potent inducer of HO-1, accelerated wound healing by decreasing the levels of oxidative stress, shown by the decrease in lipid peroxidation, and the increase in the levels of antioxidant defenses GSH, SOD, GPx, and catalase [26,76,103]. On the other hand, SnPPIX, an HO-1 inhibitor, exacerbated the production of ROS and led to a significant decrease in antioxidant enzymes [76].

Moreover, both BR and BV are known powerful antioxidants [28]. In addition, CO functions as a gaseous signaling molecule that elicits an anti-inflammatory and antioxidant response [29]. In fact, BR was shown to decrease lipid peroxidation by increasing the activity of antioxidant enzymes in rat diabetic wounds, allowing the scavenging of ROS [103]. These reports support the hypothesis that the increase in the antioxidant status of the wound is important in the management of the wound healing process in diabetes.

### 5.2. Anti-Inflammatory Properties

It is known that impaired wound healing in diabetes is associated with excess inflammation [10,104]. Several studies have shown that antioxidant treatment improves diabetic wound healing via decreasing the inflammatory response [46,99,100,103]

The immunomodulation effect of HO-1 has been reported in several immune cells. In macrophages, HO-1 induction can switch the activated M1 macrophages to the alternatively activated anti-inflammatory M2 macrophages [54,72,105], which is very important in wounds to ensure they progress through the healing stages [10]. In macrophages challenged with liposaccharide (LPS), which mimics inflammatory conditions, the over-expression of HO-1 decreased the production of inducible nitric oxide synthase (iNOS), cyclooxygenase 2 (COX2), proinflammatory cytokines levels, including tumor necrosis factor (TNF) alpha, IL-1 beta, IL-6, and macrophage inflammatory protein-1 (MIP-1), as well as increased expression of the anti-inflammatory cytokine IL-10 [105,106,107]. This effect was associated with the modulation of MAPK activities, including p38 MAPK and c-Jun NH2-terminal kinase (JNK) [73,108]. Furthermore, the lack of HO-1 in mice is associated with increased expression of IL-6, IL-1 beta, and monocyte chemoattractant protein-1 (MCP-1) [109]. It has been shown that HO-1 mediates the anti-inflammatory effect through IL-10 in mice [71]. The products derived from HO-1 activity, such as CO, BV, and BR, were also found to decrease inflammation, stimulated by LPS in macrophages, by decreasing the levels of both IL-1 beta and TNF alpha [73,108,110]. In diabetic wound animal models, the expression of pro-inflammatory cytokines, such as IL-1 beta, IL-6, and TNF alpha, are increased, and the anti-inflammatory cytokine IL-10 is decreased [26,76,84,103]. In addition, the chemokine MCP-1 and the intercellular adhesion molecule-1 (ICAM-1) were also increased in rodent diabetic wounds, contributing to an accumulation of inflammatory cells and to the buildup of an inflammatory environment [10,26,76]. The induction of HO-1 with hemin was shown to decrease the inflammatory environment as well as the accumulation of inflammatory cells in rat diabetic wounds [76]. Moreover, HO-1 induced by the activation of Nrf2 was shown to decrease the expressions of TNF alpha and IL-6 in mice diabetic wounds [34,84]. Furthermore, these anti-inflammatory effects were observed after the topical treatment with either BR, BV, or CO, in rodent diabetic wounds. This suggests that at least part of the effect of HO-1 is mediated by the products of its enzymatic activity. In addition, in rat diabetic wounds, topical treatment with BR has been shown to modulate the inflammatory wound environment with the decrease of IL-1 beta, TNF alpha, and ICAM-1, and the upregulation of IL-10 [84,111]. Similarly, BV, when applied topically to the wound site of diabetic rats, decreases IL-1 beta and TNF alpha [110]. Moreover, CO is known to promote cutaneous wound healing. Intraperitoneal administration of tricarbonyldichlororuthenium (II) dimer (CO-releasing molecule (CO-RM)-2) accelerated wound healing in rat skin through proangiogenic effects in accordance with the anti-inflammatory effects, such as the downregulation of TNF alpha and upregulation of IL-10 [112]. Moreover, CO was shown to reduce the accumulation of inflammatory cells, the expression of ICAM-1, and the activation of nuclear factor κB (NF-κB) in septic mice [113].

T-cells are important players in wound healing [114,115]. Several studies have shown the effect of HO-1 in T-cell mediated immunosuppression. Upregulation of HO-1 was shown to modulate the function of CD4^+^CD25^+^ regulatory T cells (Tregs) in immunosuppression [116]. HO-1 induction mediated by hemin was associated with the suppression of allergic airway inflammation through upregulation of CD4^+^CD25^+^ Tregs [117,118]. The transcription factor Foxp3, important in the CD4^+^CD25^+^ Treg function, was shown to induce HO-1, possibly mediating Foxp3-dependent immunosuppressive functions [119].

Furthermore, HO-1 induction was found to suppress the degranulation and proinflammatory cytokine production in mast cells in vitro and in vivo [88]. Mast cells are highly degranulated in diabetic skin, contributing to the delay in diabetic wound healing [104], suggesting that HO-1 could also improve wound healing by stabilizing mast cells in diabetic skin. 

**Figure 3:**
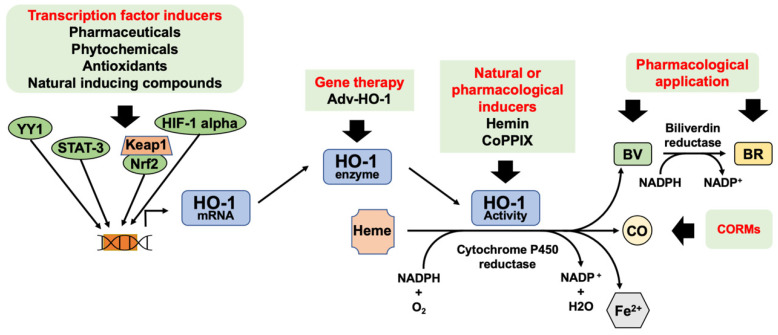
Potential therapeutic modulation of HO-1 and its enzymatic products for diabetic wound healing. HO-1 expression can be increased via activation of transcription factors or gene therapy. Natural or pharmacological inducers can be used to increase HO-1 enzymatic activity. CO, BV, and BR can be used as therapy. Legend: bilirubin (BR) biliverdin (BV), carbon monoxide (CO), CO-releasing molecules (CORMs), cobalt protoporphyrin IX (CoPPIX), heme oxygenase (HO-1), HO-1 gene transduction by adenovirus (Adv-HO-1), Kelch-like ECH-associated protein 1 (Keap1), nuclear factor erythroid 2–related factor (Nrf2).

Considering the anti-inflammatory effect of HO-1 and its reaction end products, the use of inducers of this enzyme could be an important and novel approach for translation into clinical applications to treat DFU and chronic non-healing wounds in general. The potential therapeutic modulation of HO-1 and its enzymatic products for diabetic wound healing is represented in Figure 3.

### 5.3. Cytoprotective, Migration, Proliferative and Angiogenic Properties

The cytoprotective and anti-apoptotic effects of HO-1 are well known. HO-1 is important in the inhibition of apoptosis in endothelial cells by the formation of CO, which activates the p38 MAPK pathway [70,107,120]. Furthermore, HO-1 has been shown to promote the migration of endothelial cells [9] and endothelial progenitor cells [97]. This migratory effect of HO-1 was shown to depend on a downstream target for SDF-1, the activation of vasodilator-activated phosphoprotein (VASP), a cytoskeletal-associated protein involved in motility [97,121]. In addition, HO-1 stimulates the migration of keratinocytes and improves survival under oxidative stress or hypoxia conditions, promoting proliferation in vitro [34]. Moreover, in diabetic wounds, BV was shown to increase the myofibroblast marker, alpha-smooth muscle actin (alpha-SMA), which is a very important mediator of wound closure [110].

Angiogenesis is essential in the process of wound healing. Angiogenic factors are known to be decreased in diabetic wounds, which leads to impaired angiogenesis [122,123]. HO-1 may promote angiogenesis by increasing the levels of proangiogenic agents, such as vascular endothelial growth factor (VEGF), transforming growth factor-1 (TGF-1), or stromal derived factor-1 (SDF-1) [31,97,124]. HO-1 induction improves the angiogenic potential of keratinocytes by increasing the VEGF production under high glucose conditions [125]. In addition, both CO and BV upregulate VEGF production in endothelial cells and keratinocytes in vitro [31,124]. Moreover, CO is known to stimulate angiogenesis and VEGF production through an increase of HIF-1α expression and activation of p38 MAPK kinase [87]. In fact, several studies have shown the effect HO-1 in promoting angiogenesis in wound healing animal models. In diabetic wounds, HO-1 induction by hemin or the HO-1 overexpression by gene transfer were able to increase the levels of VEGF and promote angiogenesis [76,103]. Furthermore, it has been shown that HO-1 deficient mice or the inhibition of HO-1, by SnPPIX, was associated with impaired neovascularization and ineffective wound healing and closure [34,76]. Moreover, bone marrow stem cells overexpressing HO-1 significantly promote angiogenesis and wound healing in a diabetic mouse model [126]. Studies have shown that topical treatments with BV, BR, or CO promote wound healing by stimulating angiogenesis in rodent diabetic wounds [103,110,127]. Moreover, systemic HO-1 induction contributes to an increase in circulating progenitor cells, which is important in re-endothelialization in rodents [86,128]. This is consistent with studies showing that bone marrow cells derived from HO-1 deficient mice generate fewer endothelial colony-forming cells when compared to wild-type mice [128]. HO-1 deficient mice have impaired wound healing, and this has been associated with a decrease in the recruitment of endothelial progenitor cells and capillary formation at the wound site [97]. These studies clearly demonstrate the cytoprotective, migratory, proliferative, and angiogenic properties of HO-1 and its products, thus identifying them as key players in promoting wound healing under diabetic conditions.

The induction of HO-1 expression improves diabetic wound healing. The persistent oxidative stress in diabetic wounds is decreased by the antioxidant effect of HO-1, which also promotes the increase of the expression levels of several antioxidant enzymes such as SOD, CAT, GPx, and GST. Furthermore, HO-1 decreases the levels of inflammatory markers, such as TNF alpha, IL-1beta, IL-6, iNOS, COX2, MCP-11, and MIP-1, while increasing IL-10, an anti-inflammatory cytokine, in diabetic wounds. Moreover, HO-1 was shown to induce angiogenesis in diabetic wounds by increasing the levels of proangiogenic agents such as VEGF. The decrease in oxidative stress and inflammation will promote cell viability, migration, and proliferation of skin cells for tissue regeneration. Despite the study of HO-1 in diabetic wound healing, the mechanism is not completely clarified. HO-1 was shown to decrease inflammation by promoting macrophage changes towards the M2 phenotype to increase regulatory T cells and to decrease the degranulation of mast cells. These are very important HO-1 anti-inflammatory effects that are essential in promoting diabetic wound healing. Moreover, HO-1 was found to increase the number of EPC and promote EPC-mediated angiogenesis, which is crucial for diabetic wound healing. HO-1 acts in several stages of wound healing, increasing its value as a potential therapeutic target for the treatment of DFU.

## 6. Therapeutic Potential of HO-1 Induction for DFU Treatment

Several HO-1 inducing agents include antioxidant compounds, mostly plant-based, found in the diet. These compounds activate the Nrf2 system that enhances the expression of several cytoprotective proteins such as HO-1. Among these natural compounds are resveratrol [128], curcumin [129], quercetin [130], epigallocatechin gallate [131], sulforaphane [132], and others. The efficacy of these dietary compounds at promoting wound healing when used in in vitro and in vivo models, through the induction of several protective proteins including HO-1, has led to the hypothesis that these may be used as pharmaceuticals for DFU.

Pharmaceutical compounds, such as dimethyl fumarate (DMF), have been found to activate Nrf2 [133]. DMF has shown therapeutic effects for multiple sclerosis and has been approved for clinical use [134,135]. DMF was shown to improve wound healing in diabetic mice [136], and since it is used in the clinic, it could be repurposed as a treatment for DFU. Since Nrf2 regulates multiple effector enzymes, the effects of Nrf2-inducing compounds, such as DMF, are not necessarily specific to HO-1. Further research is needed to develop safe and effective Nrf2 activator compounds for clinical use. 

Hemin, a known inducer of HO-1, has also been approved for acute intermittent porphyria treatment [137]. Hemin was shown to improve diabetic wound healing in animal models. Thus, hemin may also be a potential drug candidate for DFU treatment. 

Additionally, other pharmacological options include the use of BV/BR based therapies, which have proven to be effective in promoting wound healing in animal models [25,103,110] and/or the direct administration of CO via inhalation or CO-releasing molecules [24,29,52,108,112,113]. The exogenous application of these substances as pharmaceuticals, even at low concentrations, may not necessarily have a similar effect as the endogenous product; thus, further research is needed to evaluate their potential therapeutic effect.

## 7. Conclusions and Perspectives

Despite significant advances in the identification of targets that improve wound healing and tissue regeneration, the current therapeutic approaches to treating chronic non-healing diabetic wounds are still limited. The complex process of wound healing is highly regulated, and this regulation is difficult to achieve under diabetic conditions, in part due to the excessive oxidative and inflammatory environment which leads to impaired cell functions. Several studies have revealed that HO-1 plays a key role in all the phases of wound healing, thus identifying this marker as a promising therapeutic approach for treating chronic wounds. Moreover, the products resulting from HO-1 activity, particularly CO, BV, and BR, have also been shown to improve wound healing and are also potential therapeutic options. However, it is important to further investigate the safety of HO-1 inducers and its enzymatic end products in specific animal models and clinical trials before their applications in medical practice.

## Figures and Tables

**Table 2:** Studies supporting the role of HO-1 in wound healing.

Experimental Model	HO-1 Modulator/Agent	Findings	Refs.
HO-1 deficient mice	-	Delayed wound healing, impaired angiogenesis	[34]
Mice	SnPPIX (-)	Inhibition of HO-1 delay wound healing	
Transgenic mice	HO-1 overexpression in keratinocytes	Promote wound healing, increase angiogenesis	
Db/db mice	Adv HO-1 transduction	Promote wound healing, increase angiogenesis	
Mouse primary keratinocytes	Adv HO-1 transduction	Promote migration, increase VEGF expression	
	-		
Aortic rings from HO-1 deficient mice	CO	Impaired SDF1-mediated angiogenesis and migration	[97]
		CO reversed impaired SDF1-mediated angiogenesis in aortic rings from HO-1 deficient mice	
Diabetic rat	Hemin (+), SnPPIX (−)	HO-1 induction promotes wound healing, inhibit inflammatory cytokines, increase levels of antioxidant enzymes, and promote angiogenesis.	[26,76]

Abbreviations: carbon monoxide (CO), HO-1 transduction by adenovirus (Adv HO-1), stromal derived factor 1 (SDF1), tin protoporphyrin-IX (SnPPIX).

## Data Availability

Not applicable.
